# A new indolocarbazole derivative in melanoma and carcinoma lung in vivo treatment

**DOI:** 10.1186/s12906-021-03294-2

**Published:** 2021-04-10

**Authors:** Anna Lantsova, Irina Golubeva, Larisa Borisova, Lyudmila Nikolaeva, Lydia Ektova, Maria Dmitrieva, Olga Orlova

**Affiliations:** 1 Research Institute of Experimental Diagnostics and Therapy of Tumors, N. N. Blokhin National Medical Research Center of Oncology, Kashirskoe shosse, 24, 115478 Moscow, Russia; 2 Department of Pharmaceutical Technology and Pharmacology, Sechenov University, 8/2 Trubeckaya str., 119991 Moscow, Russia

**Keywords:** Indolocarbazole derivatives, Antitumor activity, Inhibitor, Vasculogenic mimicry, Topoisomerase, Angiogenesis

## Abstract

**Objective:**

The current scientific research direction is development of drugs with a targeted effect on malignant tumors. One of the promising groups is indolocarbazoles and their derivatives, which can initiate various tumor cell death pathways. Russian scientists from N. N. Blokhin National Medical Research Center of Oncology of the Ministry of Health of Russian Federation has developed a new experimental drug form of the original compound LCS 1269 with cytotoxic and antiangiogenic properties, blocking vasculogenic mimicry in tumor. The study aim is the experimental drug form LCS 1269 antitumor activity on models of transplantable mouse tumors B-16 melanoma and Lewis epidermoid lung carcinoma (LLC) with different routes and modes of administration.

**Material and methods:**

Female F1 hybrid mice (C_57_Bl/_6_ x DBA/2) and male and female linear mice C_57_BL/_6_ were used for management of tumor strains. Mice were obtained from N. N. Blokhin National Medical Research Center of Oncology of the Ministry of Health of Russian Federation vivarium. The antitumor effect was assessed by tumor growth inhibition (TGI) and increase of treated animal’s life span (ILS) compared to the control.

**Results:**

The experimental drug form showed high antitumor activity when administered intravenously once at doses of 100 and 120 mg/kg (TGI = 98–82% and TGI = 95–77%, respectively, ILS = 24%, *p* < 0.05) on melanoma B-16 mice. On LLC mice, the experimental drug form showed that the intravenous administration route was effective in the range of doses from 60 to 80 mg/kg with a 5 day administration regimen with an interval of 24 h. A dose of 70 mg/kg had maximum effect at the level of TGI = 96–77% (*p* < 0.05) with its retention for 20 days after the end of treatment.

**Conclusion:**

The studies have shown that the new compound LCS 1269 in the original drug form, has a pronounced antitumor activity and significantly reduces the volume of tumor mass both on melanoma B-16 and on LLC. It allows us to recommend continue the search for sensitivity of animal transplantable tumors to LCS 1269.

## Introduction

An urgent direction for scientific research is the development of drugs with a targeted effect on malignant neoplasms. One of particularly promising groups with such properties is indolocarbazoles and their derivatives, which can trigger various pathways of tumor cell death. Indolocarbazoles are known to have the ability to inhibit topoisomerases I and II involved in the control of DNA replication and transcription [1, 2].

**Fig. 1:**
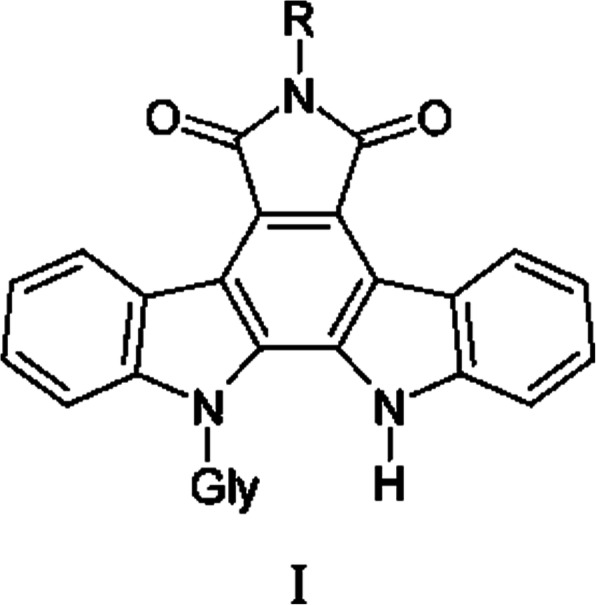
Indolo [2,3-a]pyrrolo [3,4-c]carbazole-5,7-diones-n-{12-(β-d-xylopyranosyl)-5,7-dioxo-indolo [2,3-a]pyrrolo [3,4-c] carbazole-6-yl}pyridine-2-carboxamide

Russian researchers at the N.N. Blokhin National Medical Research Center of Oncology, Ministry of Health of the Russian Federation, synthesized indolo [2,3-a]pyrrolo [3,4-c]carbazole-5,7-diones-n-{12-(β-d-xylopyranosyl)-5,7-dioxo-indolo [2,3-a]pyrrolo [3,4-c] carbazole-6-yl}pyridine-2-carboxamide (LCS 1269) [3], a new compound with cytotoxic and antiangiogenic properties, which blocks tumor vasculogenic mimicry (VM) [4] (Fig. 1).

In vitro studies LCS 1269 effectively changed VM and suppressed the angiogenesis mechanism associated with vascular endothelial growth factor (VEGF), resulting in a decreased tumor vascularization and a reduced tumor growth [5–7].

Vascular mimicry is the ability of aggressive tumor cells to form vascular-like channels, separated by the basement membrane, without the involvement of endothelial cells or fibroblasts. The formation of such structures reflects the unique ability of cells with a highly malignant cell phenotype. The appearance of the network of such channels inside a tumor has been hypothesized to support homeostasis and prevent early necrosis in a tumor [8].

Clinical trials of drugs that potentially reduce blood supply to the tumor have shown that antiangiogenic therapy is not always effective [9]. One potential cause of the survival of tumor cells is the heterogeneity of blood vessels: angiogenesis in tumors takes place in the altered extracellular matrix in the presence of uncontrolled mitogenic stimulation. It leads to the formation of defective vessels often with a broken endothelial lining. In tumor vessels, the endothelium can be absent or replaced by tumor cells. The phenomenon of VM explains the insensitivity of many malignant tumors to antiangiogenic therapy. VM is seen in almost all malignancies, and its presence correlates with the risk of metastasis and poor clinical prognosis [10].

Blockage of VM will increase the effectiveness of antiangiogenic therapy, help overcome the resistance of tumor cells to cytotoxic therapy, and prevent the transition of the tumor to a phase of more aggressive growth by reducing its plasticity. It is assumed that the antitumor effect of antiangiogenic therapy will be more pronounced if antiangiogenic agents are combined with VM inhibitors [11–17].

The relevance of our development is justified by the absence of topoisomerase I and/or II inhibitors in Russian clinical practice, while an array of chemotherapy drugs with these properties, such as irinotecan, topotecan, etoposide and teniposide, are extensively used by oncologists [14–17].

The unique mechanism of antitumor activity of LCS 1269 is explained by its dual mechanism of action, i.e. inhibition of the tumor topoisomerase and blockage of VM in the tumor. These properties justify the relevance of developing a parenteral formulation of LCS 1269. Compounds from this group are poorly soluble in water, and the creation of a drug with high bioavailability is an important and time-consuming process.

Researchers at the N.N. Blokhin National Medical Research Center of Oncology, Ministry of Health of the Russian Federation, have proposed a new experimental formulation of LCS 1269. Our research has led to the development of a stable experimental formulation containing 5 mg/ml of the active substance together with dimethyl sulfoxide (DMSO) (5%), a complex solvent (5%), ethanol (20%) and Kollidon 17PF (20%), a solubilizing and complexing agent.

This article describes a study of the specific activity of the experimental LCS 1269 formulation in transplantable mouse tumor models of B16 melanoma and Lewis lung carcinoma (LLC).

Melanoma is a leading cause of mortality from skin cancer and has a poor prognosis. Despite rapid advances in the treatment of this tumor type, the efficacy of current chemo−/targeted-therapies is still limited owing to the lack of sufficient drug accumulation in the tumor tissue and development of chemo-resistance [18–22].

Lung cancer is a malignancy with a high morbidity and mortality rate, and affected patients have low survival and poor prognosis. The therapeutic approaches for the treatment of this cancer, including radiotherapy and chemotherapy, are not particularly effective partly due to late diagnosis. Therefore, the search for new diagnostic and prognostic tools is a critical issue [23].

The aim of this study was to evaluate the antitumor activity of an original Russian antitumor indolocarbazole derivative intended for a targeted cancer therapy by assessing its antitumor effect on B16 melanoma and LLC.

## Materials and methods

### Laboratory animals

In our experiment we used (C57BL/6xDBA/2) F1 female mice and C57BL/6 male and female mice, with the latter being used to inoculate tumor cells. The mice were obtained from the vivarium at the N.N. Blokhin National Medical Research Center of Oncology. The mice were kept in conventional conditions on briquetted feed and provided constant access to water [24].

The animals were divided into experimental (*N* = 6–8) and control groups (untreated, *N* = 10). All animal protocols were approved by the Institutional Animal Care and Use Committee at the IBMC.

### Models of tumor growth

The antitumor activity of the study compound LCS 1269 was evaluated on two transplantable murine tumors, i.e. B16 melanoma and LLC.

B16 melanoma is a non-immunogenic tumor that has been maintained in C57BL/6 mice. The tumor arose spontaneously in 1954 in the skin at the base of the ear of a C57BL/6 mouse. This cancer cell line was obtained in 1975 from the frozen tumor bank (USA). It has a heterogeneous cell population, including both highly pigmented sites and areas with little or no melanin. It metastasizes to the lungs in 60–90% of cases, and sometimes to the liver and spleen. The average life span of animals with this tumor is 25–30 days.

LLC grows rapidly, metastasizes to the lungs in 100% of cases, and is selectively sensitive to certain groups of anticancer drugs. LLC occurred spontaneously in a C57BL6 mouse in 1951. This cancer cell line was obtained from the U.S. National Cancer Institute in September 1973. The tumor consists of polymorphic cells, most of which are round in shape. A significant part of the cell is occupied by a nucleus with large nucleoli and large condensed chromatin bodies. LLC has been maintained in C57BL/6 mice. The average life span of animals with this tumor is 24 days.

The cancer cell lines were obtained from the cancer cell line bank at the N.N. Blokhin National Medical Research Center of Oncology. The B16 melanoma cell line was maintained in C57BL/6 female mice, who received subcutaneous grafts every 12–14 days, according to the standard procedure of serial grafting. The LLC cell line was maintained in C57BL/6 males, who received intramuscular grafts every 12–14 days [25]. Experiments were started from the fourth passage of the tumors in vivo. In our experiment, tumor transplantation was performed according to the standard method used at the N.N. Blokhin National Medical Research Centre of Oncology. Tumors were transplanted to (C57BL/6xDBA/2) F1 hybrid mice. A total of 0.5 ml (50 mg) of tumor cell suspension (a 1/10 dilution in medium 199) was injected subcutaneously (s.c.) in the right axillary region [11, 12]. Treatment was started 48 h after transplantation [25, 26].

### Study compound

The experimental formulation of the study compound contained LCS 1269 5 mg, DMSO 5%, Kollidon 17PF 20%, ethanol 20%, and water up to 100%.

### Dosing regimens and routes of administration

The antitumor effect of LCS 1269 on B16 melanoma and LLC was studied by administering this agent in an experimental injectable formulation through different routes of administration using the following dosing regimens:

- intravenously (i.v.) daily for 5 days (24 h × 5) at doses of 60, 70 or 80 mg/kg (total doses 300, 350 or 400 mg/kg, respectively);
- i.v. at a single dose of 100 or 120 mg/kg (for B16 melanoma-bearing mice) and 150 or 180 mg/kg (for LLC-bearing mice);
- intraperitoneally (i.p.) daily for 5 days (24 h × 5) at a dose of 60 mg/kg (total dose 300 mg/kg);
- two i.p. doses of 100 or 120 mg/kg at a 96-h interval (total doses 200 or 240 mg/kg, respectively);
- s.c. daily for 5 days (24 h × 5) at a dose of 60 mg/kg (total dose 300 mg/kg);
- two s.c. doses of 100 or 120 mg/kg at a 96-h interval (total doses of 200 or 240 mg/kg, respectively);
- two oral doses of 120 mg/kg at a 96-h interval (total dose of 240 mg/kg);
- per os daily for 5 days (24 h × 5) at doses of 130, 150 or 200 mg/kg (total doses 650, 750 or 1000 mg/kg, respectively).

These doses were selected based on the in vitro and in vivo pre-screening assessment of the antitumor activity of LСS 1269 substance, as well as the dose regimens used for LСS 1208, another experimental drug of this class [27].

#### Criteria for assessing antitumor activity

The following criteria for assessing antitumor activity were applied: tumor growth inhibition (TGI, %) and percentage increase in life span (ILS, %) of the test animals compared with the control animals [25, 26].

The antitumor effect was assessed by measurements of tumor volume, which were done every 3–4 days, depending on tumor growth rate.

The tumor volume was calculated by multiplying its maximum dimensions in three orthogonal directions (length, *l*; width, *w*, height, *h*). This parameter was calculated for all animals.

TGI was calculated using the following formula (1):

1 $$ \mathrm{TGI}\left(\%\right)=\left(\mathrm{Vc}-\mathrm{Ve}\right)/\mathrm{Vc}\ \mathrm{x}\ 100, $$

where: TGI – tumor growth inhibition;

Vc – mean tumor volume in the control group (mm^3^);

Ve – mean tumor volume in the experimental group (mm^3^).

ILS was calculated using the following formula (2):

ILS (%) = (MLSc - MLSe)/ MLSc × 100, (2).

where: ILS – increase in life span;

MLSc – mean life span of the animals in the control group (days);

MLSe – mean life span of the animals in the experimental group (days).

The following minimal efficacy criteria were used: TGI ≥50% and ILS ≥25% for animals with a solid tumor. The effective dose was defined as the dose of the study compound resulting in TGI ≥70% persistent for at least 7 days post-treatment [25, 26].

#### Evaluation of treatment tolerability in tumor-bearing mice

The animals were monitored until death. The condition of the animals was evaluated by daily visual inspections. The tolerability of this chemotherapeutic compound was assessed by the condition and behavior of the mice. The toxicity of the regimens and doses used in the study was assessed by time to death in the experimental group compared with that in the control group.

The statistical analysis was done using the Statistica 6.0 software (StatSoft, Tulsa, OK), with study data being analyzed by the Student’s t-test. The differences were considered significant at *p* ≤ 0.05.

## Results

The search for active anticancer ingredients starts with the study of chemical compounds obtained by a random or streamlined synthesis, which is followed by a two-stage initial selection, including in vitro pre-screening and in vivo screening of active substances. If a compound demonstrates an antitumor activity, then it undergoes the standardization process, and the possibility for creating a dosage form is assessed. If a dosage form is created, it is then evaluated in in-depth preclinical and clinical studies. Evaluation of the efficacy and assessment of the specific activity of the new drug product are performed as one of the stages of preclinical studies. For anticancer drugs, this includes search for sensitive tumors, an identification of the most effective mode of administration and selection of the therapeutic dose range by comparing doses causing death of animals and non-lethal doses providing an antitumor effect. Our research was focused on this stage.

This article presents data about studying tumors sensitive to a new experimental dosage form of LCS 1269, which were obtained from experiments performed on models of transplantable murine tumors (B16 melanoma and LLC).

### Antitumor efficacy of the experimental formulation of LCS 1269 on melanoma B16 mice

**Fig. 2:**
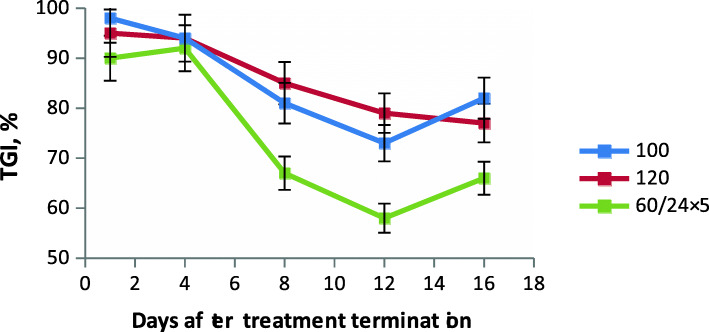
Correlation between TGI in murine B16 melanoma and i.v. dose of LCS 1269

The antitumor efficacy of LCS 1269 was studied by administering this agent in an experimental formulation to mice with B16 melanoma through different routes of administration using different dosing regimens (Table 1, Fig. 2).

**Table 1:** Antitumor efficacy of the experimental formulation of LCS 1269 for B16 melanoma

Route of administration	Dose (mg/kg)/ interval (h) x number of administrations	TGI (%) Days post-treatment					ILS (%)	Death of animals (%)
		1	4	8	12	16		
i.v.	60/24х5	90*	92*	67*	58*	66*	–	17
	100х1	98*	94*	81*	73*	82*	24*	0
	120х1	95*	94*	85*	79*	77*	4	0
i.p.	60/24х5	95*	98*	83*	76*	73*	26*	0
per os	120/96х2	66*	67*	43	47	64*	9	0
s.c.	60/24х5	83*	78*	45	41	60*	25*	0
	120/96х2	78*	75*	42	32	63*	–	50

**Note:** * *р* < 0.05 compared with control

When administered in its experimental formulation at a daily dose of 60 mg/kg for 5 days (total dose 300 mg/kg), LCS 1269 showed a pronounced long-term antitumor effect with TGI 90–66% (*p* < 0.05 compared with control), which was maintained until day 16 post- treatment. However, 17% of the animals in this group showed signs of toxicity and died. Toxicity was assessed by mean spleen weight in the treatment group (mean weight 95.2 ± 46.1 mg) compared to the control group (mean weight 109.3 ± 37.9 mg). Normal spleen weight in healthy animals is about 190.2 ± 35.3 mg.

After single i.v. administration of LCS 1269 at 100 mg/kg or 120 mg/kg, the highest antitumor effect was observed with TGI 98–82% and 95–77% (*p* < 0.05), respectively, which persisted until day 16 post-treatment. No animal deaths were observed.

When administered at a dose of 60 mg/kg i.p. under a 24-h × 5 regimen, LCS 1269 showed a high long-term antitumor effect with TGI 95–73% (*p* < 0.05), which persisted until day 16 post-treatment, and ILS 26% (*p* < 0.05). No deaths were observed.

Administration of LCS 1269 at two oral doses of 120 mg/kg (total dose 240 mg/kg) at a 96-h interval resulted in a moderate antitumor effect, with TGI persisting at 66–64% (*p* < 0.05) until day 16 post-treatment. No deaths were reported. No increase in life span was seen.

Daily s.c. administration of LCS 1269 at a dose of 60 mg/kg (total dose of 300 mg/kg) for 5 days produced a high short-term antitumor effect with TGI 83–60% (*p* < 0.05), which persisted until day 4 post-treatment; a minimal effect (TGI 45–60%, *p* < 0.05) was observed until day 16 of observation. The second minimal efficacy criterion (ILS 25%) was also reached (*p* < 0.05). No deaths of animals were reported. When administered at two s.c doses at a 96-h interval, LCS 1269 produced toxicity at the higher dose (120 mg/kg), despite the lower course dose of 240 mg/kg, and caused the death of 50% of the animals in this group.

### Antitumor efficacy of the experimental formulation of LCS 1269 on LLC

**Fig. 3:**
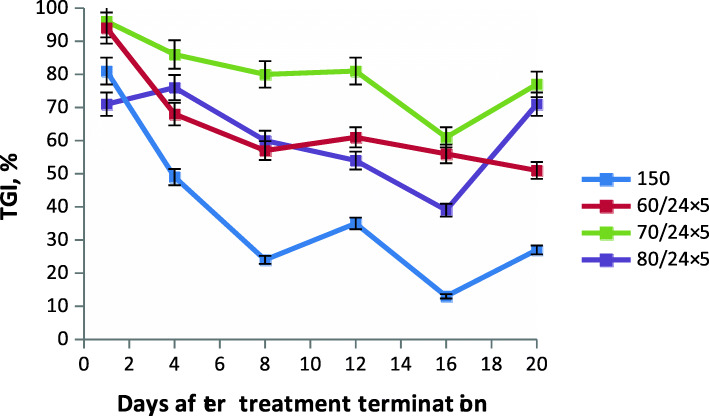
Correlation between TGI in murine LLC and i.v. dose of LCS 1269

The efficacy of LCS 1269 for LLC was studied in a wide range of doses (60 mg/kg to 200 mg/kg), using different routes of administration and dosing regimens (Table 2, Fig. 3).

**Table 2:** Antitumor efficacy of the experimental formulation of LCS 1269 for LLC

Route of administration	Dose (mg/kg)/ interval (h) x number of administrations	TGI (%) Days post-treatment						ILS (%)	Death of animals (%)
		1	4	8	12	16	20		
s.c.	60/24 × 5	78*	78*	66*	61*	45	58*	34*	0
	100/2 × 96	80*	75*	69*	64*	64*	68*	15*	0
	120/2 × 96	86*	79*	72*	71*	64*	63*	19*	0
per os	130/24 × 5	64*	56*	52*	31*	35*	24	–	0
	150/24 × 5	69*	64*	61*	44*	42*	43*	13*	0
	200/24 × 5	45*	56*	48*	41*	28	45*	–	0
i.v.	60/24 × 5	94*	68*	57*	61*	56*	51*	11*	0
	70/24 × 5	96*	86*	80*	81*	61*	77*	8*	0
	80/24 × 5	71*	76*	60*	54*	39*	71*	9*	0
	150 × 1	81*	49*	24	35	13	27	–	0
	180 × 1	–	–	–	–	–	–	–	86
i.p.	60/24х5	63*	60*	52*	37	39	41	23*	0
	100/2 × 96	53*	53*	49*	47*	36	47*	27*	0
	120/2 × 96	61*	73*	68*	61*	66*	67*	–	0

**Note:** * *р* < 0.05 compared with control

A five-day course of daily s.c. LCS 1269 (60 mg/kg at a 24-h interval) given in its experimental formulation was associated with TGI 82% immediately post-treatment, which persisted at 58% (*p* < 0.05 compared with control) until day 20 of observation, and with ILS 34%. Administration of two s.c. doses of 100 mg/kg or 120 mg/kg at a 96-h interval resulted in a high antitumor effect, i.e. TGI ranging from 80 to 68% to 86–63% (*p* < 0.05) over a period of 20 days (Table 2).

Evaluation of the antitumor activity of the study compound against LLC was also performed after a five-day course of daily oral LCS 1269 given in its experimental formulation at a 24-h interval. It showed that administration at doses of 130 mg/kg and 150 mg/kg was associated with TGI 64–52% and 69–61% (*p* < 0.05), respectively, which persisted until day 8 post-treatment (Table 2).

The data obtained in this study showed that the highest antitumor effect on murine LLC was achieved with a five-dose course of LCS 1269 given in its experimental formulation at daily i.v. doses of 60–80 mg/kg (total dose 300–400 mg/kg, respectively). The maximum effect—TGI 96% (*p* < 0.05) persistent at 77% until day 20 post-treatment—was achieved with the dose of 70 mg/kg. A single i.v. dose of 180 mg/kg produced toxicity-related deaths of 86% of the mice in this group 4 days after treatment. Toxicity was assessed by the mean spleen weight in the treatment group (mean weight 33.3 ± 11.4 mg) compared with the control (mean weight 110.4 ± 53.7 mg).

Comparison of two i.p. dosing regimens—five doses at a 24-h interval and two doses at a 96 h interval—showed that the highest antitumor effect was achieved with two doses of 120 mg/kg given at a 96-h interval. TGI was 61% and persisted at 67% (*p* < 0.05) until day 20 post-treatment.

## Discussion

Thus, evaluation of the antitumor activity of the LCS 1269 experimental formulation in transplantable murine tumors showed that it exerted a high antitumor effect against B16 melanoma when administered at single i.v. doses of 100 mg/kg or 120 mg/kg (i.e. TGI 98–82%, ILS 24% and TGI 95–77%, respectively; *p* < 0.05); as well as at i.p. doses of 60 mg/kg at 24-h intervals for 5 days (TGI 95–73%, ILS 26%, *p* < 0.05). This effect lasted for 16 days after the end of treatment.

In LLC-bearing mice, the most pronounced antitumor effect was observed after a five-day course of i.v. LCS 1269 given at daily doses of 70 mg/kg at a 24-h interval, i.e. TGI 96–77% (*p* < 0.05), which persisted until day 20 post-treatment.

As shown in Tables 1 and 2, deaths were reported in mice who received the drug at the highest total doses (240 mg/kg and 300 mg/kg) and at the highest single dose (180 mg/kg), i.e. doses at which exposure to the active substance of LCS 1269 was associated with general toxicity rather than an antitumor effect. This was supported by a decreased mean spleen weight in the experimental group compared with the control group of healthy animals (mean spleen weight in mice 190 mg).

The results of this experimental study are comparable with other authors’ data about the antiproliferative activity of different indolocarbazole derivatives [12–17].

This supports the necessity and urgency of further preclinical studies of the new experimental formulation of LCS 1269. These data also suggest that this formulation may be effective against a wider range of transplantable animal tumors.

## Conclusion

Studies have shown a marked antitumor effect of the new LCS 1269 drug product in its original formulation against both murine B16 melanoma and LLC. It was also proven to reduce significantly the tumor volume, which suggests a further search for transplantable animal tumors sensitive to LCS 1269.

## Data Availability

All data generated or analyzed during this study are included in this published article.

## Abbreviations

DMSO: Dimethylsulfoxide
ip: Intraperitoneally
iv: Intravenously
ILS: Increase in life span
LLC: Lewis lung carcinoma
TGI: Tumor growth inhibition
VM: Vasculogenic mimicry
